# The Effect of Physical Activity Interventions on Executive Function in Overweight and Obese Adults: A Systematic Review

**DOI:** 10.3390/biomedicines12122724

**Published:** 2024-11-28

**Authors:** María Elena Chávez-Hernández, Lizbeth De La Torre, Luis Miguel Rodríguez-Serrano, Marina Wöbbeking-Sánchez

**Affiliations:** 1 Facultad de Psicología, Universidad Anáhuac México, Universidad Anáhuac Avenue #46, Lomas Anáhuac, Huixquilucan 52786, Mexico; elena.chavez@anahuac.mx; 2 Facultad de Psicología, Universidad Pontificia de Salamanca, Calle de la Compañía, 5, 37002 Salamanca, Spain; ldelatorrelo@upsa.es; 3 Facultad de Psicología, Universidad de Salamanca, Avenida de la Merced, 109, 37005 Salamanca, Spain

**Keywords:** physical activity, executive function, overweight, obesity, adults

## Abstract

**Background/objectives**: Overweight and obesity are global public health problems associated with chronic disease and mental health. Physical activity (PA) is essential throughout a person’s life; an active lifestyle helps people to live healthier lives and improve their functional and mental abilities, such as executive function (EF). This systematic review aimed to analyze the evidence on the effects of PA on EF in overweight and/or obese adults (≥18 years old). **Methods**: Records from the PubMed, ScienceDirect, and JSTOR databases were searched and, following the PRISMA guidelines, seven studies were included in the present systematic review. Risk of bias was assessed using the National Institute of Health Quality Assessment Tool for Observational Cohort and Cross-Sectional Studies. Results from the studies included indicate that acute, short-term, and long-term PA interventions are an effective strategy to improve inhibitory control, working memory, and processing speed in overweight and obese adults. Furthermore, evidence indicates that EF can be effective as a measure to predict adherence to PA programs and weight loss. **Conclusions**: Exercise and physical activity interventions are a promising therapeutic strategy to promote weight loss and improve EF in adults with overweight and obesity. Additionally, EF may be further explored as a predictor of healthy aging due to the choices made throughout life and the long-term benefits that result.

## 1. Introduction

Overweight and obesity are global public health problems that result from an imbalance of energy consumption and energy expenditure, with a multifaceted nature noting genetic, behavioral, and environmental variables as major contributors [1]. In this regard, sedentarism, lower physical activity (PA), and the excess consumption of palatable foods are risk factors that have been identified as leading to overweight and obesity [2]. Additionally, overweight and obesity have been associated with chronic diseases such as cardiometabolic disease, mechanical disease, cancer, and mental health [3,4]. PA has been shown to be an effective behavioral and environmental intervention strategy that can empower young and older adults to make positive lifestyle choices and promote healthy body weight [5].

Human beings are in a constant state of physical, psychological, and emotional change, and their health and physical condition depend on the actions they take in their daily lives and on how they live from youth to old age. For this reason, a healthy life is essential at all stages of a person’s life, even more so in old age, when a greater number of limitations appear because the body loses various physical and intellectual capacities, which become more noticeable over time [6].

Del Conde et al. [7] point to a decline in the need for physical movement and activity in daily routines that predates the evolutionary biological adaptation of humans. This dissonance between what the body was designed to do and what today’s society requires of us has raised significant concerns about the physical and psychological well-being of society as a whole, as sedentary lifestyles not only affect physical health by increasing the risk of diseases such as type 2 diabetes, cardiovascular disease, and certain cancers but can also negatively affect mental health by promoting the onset of disorders such as depression and anxiety [8,9].

The current trend towards less active lifestyles is particularly marked in urban areas, where the conveniences of modern life (such as public transport, fast food, and technological entertainment) have led to a sharp decline in daily PA levels [9]. Additionally, physical inactivity is a major risk factor for mortality, so its continued and regular practice reduces the risk of physical and cognitive problems and non-communicable diseases [10]. The risk of death is 30% higher in inactive people, so regular PA is associated with better mental health [11,12]. In this regard, PA is recommended to improve both physical fitness and health-related quality of life (HRQOL) at all stages of life. An active lifestyle has been shown to help people live healthier, more independent lives and improve their functional and mental abilities. Physical fitness and HRQOL are closely related, as they promote the maintenance of global levels of functioning and a satisfactory aging process, the aim of which is to maintain the autonomy and independence of adults [13].

On the other hand, executive functions (EFs) are advanced cognitive processes that involve planning, initiating, shifting, monitoring, and inhibiting behavior. EFs play an important role in our daily lives, allowing us to focus our attention on specific tasks, successfully solve problems, and plan for the future, through the four key components of EF: inhibition, working memory, cognitive flexibility, and planning [14].

As a human grows, so do the EFs, showing variable developmental and aging profiles, with prolonged development into early adulthood and a decline into old age that is associated with structural and functional changes in the prefrontal cortex [15]. EFs begin to develop early in infancy, with basic skills needed for EFs emerging before three years of age, and more specific skills developing into early childhood. With childhood and adolescence being periods of extensive brain maturation and reorganization, they are shaped by multiple genetically based biological processes in dynamic interaction with the environment [16]. Additionally, this cortical maturation is reflected in cognitive improvements from early childhood to young adulthood, with the development of basic functions such as sensorimotor processes preceding complex functions such as top-down control of behavior [17].

Cognitive performance is thought to peak in young adulthood, with declines beginning as early as 20 or 30 years of age, including declines throughout adulthood in EF components such as working memory, which may also be related to different environmental contexts [18]. Evidence has shown that EFs are key to facilitating planning and maintaining goal-directed behaviors by facilitating self-regulatory mechanisms [19]. In this regard, individuals with obesity often report difficulties controlling eating behaviors, despite a desire to lose weight successfully [20]. Furthermore, in overweight/obese individuals, a theoretical association has been stated between excess weight and impairments in EF [21]. Additionally, cognitive flexibility seems to be a key mechanism to enable individuals to capitalize on interruptions in unhealthy habits by adjusting their behavior in line with their weight loss goal rather than persisting with unhealthy habits [22].

In this regard, studies show that individuals with food addiction to palatable foods have altered connectivity in reward dopaminergic pathways [23] and that overconsumption of palatable foods can be a risk factor in the development of overweight and obesity [24]. Additionally, EFs are most strongly linked to disinhibitory eating behaviors [25,26,27]. Franco-García et al. [28] show that older adults who are sedentary with a low level of education, and who are overweight, are at the highest risk of developing cognitive impairment. In this regard, PA has been identified as an effective strategy to improve EF in children and adolescents with overweight and obesity [29,30,31]. Nevertheless, there are few studies focusing on adulthood, and given that overweight and obesity have been shown to impair EF, it is important to systematically analyze changes associated with PA and exercise in adults with these conditions. Overweight and obesity indices among adults have become a significant global health concern, given that these conditions represent an increased risk of cognitive decline in this population. Furthermore, it is important to understand the effect of PA interventions in overweight and obese adults given that it may be helpful to inform and design them. Therefore, the aim of the present systematic review was to identify the effect of PA interventions on EF in adults with overweight and obesity.

## 2. Materials and Methods

### 2.1. Information Sources and Search Strategy

Our research question followed the PICO (population, intervention, comparison, outcome) format, including the following elements: Population: overweight and obese adults (≥18 years old); Intervention: exercise; Comparison: pre-post measure or comparison groups (control and/or different exercise intensities); Outcome measure: executive function evaluation.

A systematic search was directed following PRISMA guidelines for systematic reviews and meta-analyses [32,33]. Three databases were searched: PubMed, ScienceDirect, and JSTOR. The following terms were used to conduct the search: “physical activity”, “exercise”, “obese”, “overweight”, “executive function”, and “adults”. These descriptors were searched in the title and abstract fields. The registration code is INPLASY2024100078.

English-language scientific articles were included without restrictions on publication date. The following inclusion criteria were determined: peer-reviewed articles, longitudinal and/or cross-sectional studies, randomized and/or observational studies, and studies including comparison groups and/or pre-post measures. Articles were not included under the following exclusion criteria: review articles, systematic review and/or meta-analysis articles, studies including participants with comorbidities (diabetes, hypertension, etc.), and studies where full text was not available.

### 2.2. Data Extraction and Risk of Bias Assessment

PRISMA guidelines were followed for identifying and extracting the articles for this systematic review. The search for and extraction of articles were performed by two authors (R-S and C-H). Title and abstract screening, as well as full-text screening, were made by consensus of all authors. Additionally, the risk of bias was assessed by two reviewers (R-S and C-H) using the National Institute of Health (NIH) Quality Assessment Tool for Observational Cohort and Cross-Sectional Studies and reviewed by two authors (W-S and D-L), which includes the following fourteen questions:

Q1. Was the research question or objective in this paper clearly stated?

Q2. Was the study population specified and defined?

Q3. Was the participation rate of eligible persons at least 50%?

Q4. Were all the subjects selected or recruited from the same or similar populations (including the same period)? Were inclusion and exclusion criteria for being in the study prespecified and applied uniformly to all participants?

Q5. Was a sample size justification, power description, or variance and effect estimates provided?

Q6. For the analyses in this paper, were the exposure(s) of interest measured before the outcome(s) being measured?

Q7. Was the timeframe sufficient so that one could reasonably expect to see an association between exposure and outcome if it existed?

Q8. For exposures that can vary in amount or level, did the study examine different levels of the exposure as related to the outcome (e.g., categories of exposure, or exposure measured as a continuous variable)?

Q9. Were the exposure measures (independent variables) clearly defined, valid, reliable, and implemented consistently across all study participants?

Q10. Was the exposure(s) assessed more than once over time?

Q11. Were the outcome measures (dependent variables) clearly defined, valid, reliable, and implemented consistently across all study participants?

Q12. Were the outcome assessors blinded to the exposure status of participants?

Q13. Was the loss to follow-up after baseline 20% or less?

Q14. Were key potential confounding variables measured and adjusted statistically for their impact on the relationship between exposure(s) and outcome(s)?

## 3. Results

### 3.1. Search Results

**Figure 1:**
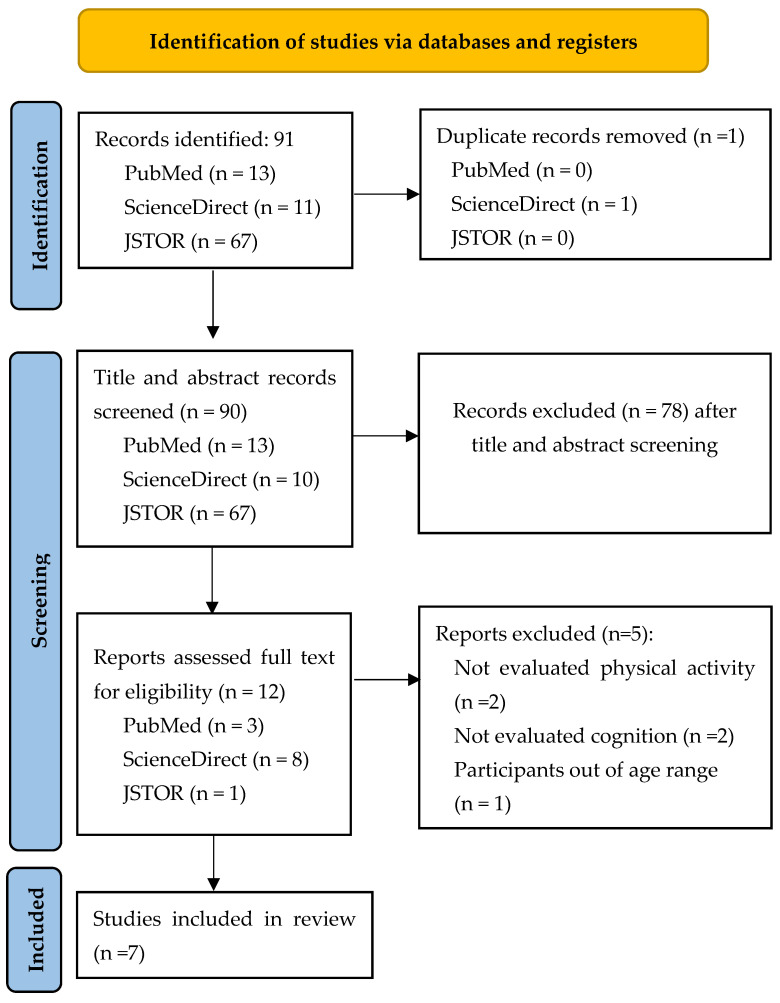
Flow diagram of study selection.

A total of 91 studies were identified from three databases: PubMed, ScienceDirect, and JSTOR. The duplicates were removed, and 90 records were included for title and abstract screening. Of these, 12 were included in their full text for screening. After full-text screening, 7 studies were eligible for inclusion in the systematic review (Figure 1).

Of the studies included, two (28.6%) had a long-term 6-to-12-month intervention [34,35], while two (28.6%) had a short-term 4-to-6-week intervention [36,37], three (42.8%) had acute exercise sessions [38,39,40]. Furthermore, 4 studies (57.1%) included both moderate- and high-intensity exercise interventions [34,35,36,38], one study (14.3%) included aerobic and anaerobic exercise [37], one (14.3%) only moderate-intensity [40], and one (14.3%) only high-intensity intervention [39].

Regarding the age of participants, one study (14.3%) included adults 18 to 70 years of age [34], three (42.8%) included young adults [36,37,38], one (14.3%) included middle age and older adults [40], and two (28.6%) included young and middle-aged adults [35,39].

As for EF changes, five studies (71.4%) evaluated changes in EF after a PA intervention [35,36,38,39,40], while two studies (28.6%) evaluated EF as a predictor for weight loss and moderate-to-vigorous exercise at the end of intervention [34,37].

### 3.2. Risk of Bias Assessment

Table 1 shows the results from the Risk of bias assessment, performed using the NIH Quality Assessment Tool for Observational Cohort and Cross-Sectional Studies (https://www.nhlbi.nih.gov/health-topics/study-quality-assessment-tools, accessed on 7 September 2024)

### 3.3. Effect of Acute Exercise Sessions on Executive Function

The results from the studies included are presented in Table 2. Studies show that a single 16 min session of aerobic exercise is sufficient to improve inhibitory control, working memory, and processing speed in lean and non-glucose-tolerant obese adults [39]. In addition, in older adults, a single 30 min walk at moderate intensity has been shown to improve executive function and, when combined with 30 min of light intermittent sitting, to improve working memory [40]. Furthermore, in overweight inactive young male adults, Quintero et al. [38] demonstrated that in overweight young male adults, progressive resistance and HIIT training combined improve cognitive inhibition and attention capacity compared to progressive resistance training alone.

### 3.4. Effect of Short-Term Exercise Interventions on Executive Function

When evaluating the effect of short-term exercise interventions on executive function in overweight and obesity, 6-week moderate-intensity or high-intensity training in young adults has been shown to improve executive function. On the other hand, Xu et al. [37] demonstrated that EF can also be used as a predictor of weight loss during a 4-week intervention program including aerobic and anaerobic exercise, where better performance in the Stroop task is correlated with greater weight loss in young adults.

### 3.5. Effect of Long-Term Exercise Intervention on Executive Function

Peven et al. [35] demonstrated that a 12-month intervention combining diet and high-intensity exercise significantly reduced reward sensitivity and improved reward processing.

### 3.6. Executive Function as a Predictor for Weight Loss

EF can also be used as a predictor for weight loss and physical activity. In this regard, Xu et al. [37] demonstrated that better EF performance was correlated with greater weight loss during a 4-week intervention program including aerobic and anaerobic exercise, while Butryn et al. [34] also showed that better planning, inhibitory control, and flexibility significantly predicted more minutes per week of moderate-to-vigorous PA after a 6-month intervention.

### 3.7. Effect of Improving Executive Functions Related to BMI and PA Intervention Program

The average body mass index (BMI) of the participants in the included trials was 32.8%, with a minimum of 28.1% and a maximum of 36.16%. In the results, no significant differences were found in the association between BMI and improvement in executive function, as the improvement was consistent regardless of body mass index to a greater or lesser extent. What did show a difference was the type of intervention, with more significant results in the functions of processing speed, attention, and memory in those interventions that combined moderate-to-vigorous-intensity physical activity with dietary changes, and where these changes were sustained for more than 4 weeks, compared with less consistent results that were not sustained in the long term when physical activity programs did not last more than 4 weeks and promoted low-intensity activities without changes in diet.

## 4. Discussion

Prior research has suggested that obesity/overweight may be associated with deficits in EF throughout life, and it has been found that obese participants showed broad impairments in EF [21]. Nowadays, obesity has become a worldwide public health issue, and its impact on EF has been consistently reported [41]. The present systematic review aimed to analyze evidence showing the effect of physical activity on executive function in overweight and/or obese adults. To achieve this, the PICO research question considered the following elements: Population: overweight and obese adults (≥18 years old); Intervention: exercise or physical activity; Comparison: pre-post measure or comparison groups (control and/or different exercise intensities); Outcome measure: executive function evaluation. Evidence from the included studies indicates that PA interventions, from acute to long-term durations, have benefits in improving EF for those with overweight and obesity. In this regard, the practice of physical activity plays an essential and relevant role in the development of health with its benefits for executive function in adults having been analyzed [42,43,44].

Some studies have reviewed the correlation evidence for PA in weight and adiposity loss, prevention of weight, and adiposity gain. Research suggests that even >150 min of at least moderate-intensity aerobic activity per week can prevent weight and adiposity gain; nonetheless, an upper range of PA is required to avoid weight regain after weight loss [45]. The evidence on whether high-intensity physical activity or resistance exercise is better for weight management in the short and long term is still uncertain. Thus, exercise professionals should advise that metabolic and cardiovascular health benefits can be achieved with physical activity at any weight and regardless of weight change [46].

The practice of PA has a relevant impact on the development of executive functions since there are certain cognitive demands inherent in the motor stimuli implemented in the practice of different PAs [47]. In this regard, the studies included show that a single high-intensity exercise session improves working memory and attention, which shows the high impact that PA has on improving EF in adults with obesity [39].

On the other hand, a significant association has been found between moderate PA and improved performance on tests of EF, especially in older adults aged 76–85. Study results show that PA is an important factor in maintaining EF in older adults with metabolic syndrome [48]. The present review shows evidence that in overweight inactive young adults, short-term high-intensity PA improves cognitive inhibition and attention capacity in overweight/inactive young adults [38], and also that both moderate- and high-intensity PA shows benefits for improving EF in obese sedentary young adults [36]. Regarding the effects of long-term PA interventions, evidence from the present review indicates that a six-month PA program improves planning, inhibitory control, and long-term flexibility [34].

Furthermore, a 12-month diet and moderate or intensive PA intervention significantly improves the overall performance of EF, compared to the short-term improvements in cognition found with only dietary changes. This highlights the importance of including PA to consolidate improvements in cognition over the long term in adults with obesity [35]. In this regard, Castillo et al. [49] conducted a study on an adult population over 65 years of age with impaired executive functions. The results showed that 36.21% of the participants between the ages of 65 and 75 had severe executive function impairment, whereas an increase in moderate PA was observed in the group between the ages of 76 and 85 (11.02%), suggesting a positive relationship between PA and the maintenance or improvement of EF. Likewise, Martín-Martínez et al. [50] suggest that higher levels of physical activity increase skills such as planning, cognitive flexibility, and/or inhibitory control, which are characteristics of EF. This is reflected in the results of this study, which show that those with higher levels of physical activity have higher average EF.

Xu et al. [37] found that a better performance on the Stroop task performance, which evaluates EF, correlated with greater weight loss in overweight and obese young adults. In their study, a reduced reaction time interference predicted a greater weight loss percentage; furthermore, overweight and obese individuals with a greater hemodynamic response in the left ventrolateral and bilateral dorsolateral prefrontal cortex lose more weight during short-term fitness activities. Additionally, Butryn et al. [34] found that performance in an EF evaluation battery significantly predicted moderate-to-vigorous PA after 6 months, where participants without rule violations had more minutes per week than those with rule violations. Butryn et al.’s [34] results are consistent with what Eichen et al. [51] report, supporting that lower EF may be associated with attenuated weight loss following BWL, and targeting EF in treatment could improve outcomes. Interestingly, both Butryn et al. [34] and Xu et al. [37], in contrast with most studies included, analyzed EF as a predictor of weight loss and PA, which is an interesting approach given that deficits in EF can become a barrier to weight-related behavior change; individuals with poorer EF are less likely to adhere to PA interventions/eating habit changes or prescriptions due to poorer inhibitory control.

Therefore, it is important to consider the importance of PA not only as a pathway to general well-being but also as a way to prevent cardiovascular disease, which can lead to cerebrovascular events and later cognitive impairment at an earlier age [52]. In this sense, some studies suggest that vigorous physical activity mediates less than 10% of the association between obesity and cognitive decline in middle-aged and older adults. This is an important reason to develop further studies that explore the potential factors associated with the obesity–cognitive paradox [53]. 

## 5. Conclusions

The present systematic review shows compelling evidence of the benefits of PA in EF ranging from single sessions to long-term interventions, given that the studies included show improvement in at least one EF variable, indicating a consistency of the benefits of PA on EF in overweight and obese adults, as well as the relevance of exercise and its benefits in overweight and obese adults. Furthermore, the present systematic review helped identify how exercise and PA interventions may present as a promising therapeutic strategy not only to promote weight loss but also to improve EF and overall well-being in this population, providing evidence-based recommendations for clinical practice and public health interventions.

Most of the studies included in this review agree with the improvements coming from PA interventions and a healthy lifestyle, but there are also outcomes focusing on targeting EF for weight loss in adults; therefore, it might also be interesting to further research EF as a predictor for healthy aging due the decisions made through the life span and the long-term benefits coming from it. It is important to note that a potential limitation may be that most studies focus on acute and short-term interventions, which raises questions regarding the long-term effects of these PA interventions on EF in overweight and obese adults. Additionally, most of the included studies focus on young and middle-aged adult groups, with little attention on the effects on overweight and obese older adults. Furthermore, another potential limitation is the heterogeneity of the study designs and PA interventions of the included studies in terms of durations and intensity, as well as the heterogeneity of the tasks used to evaluate EF. In this regard, we recommend future systematic reviews focus on specific EF tasks and interventions with specific PA characteristics (e.g., intensity, duration, etc.), as well as evaluate further the long-term effects of PA interventions on EF in this population. Despite these potential limitations, evidence from the present systematic review indicates that these non-pharmacological alternatives could be useful in assessing public health conditions that are increasingly present in younger people, raising the worrying scenario of more people being susceptible to pathological aging, and providing evidence-based recommendations to address these concerns.

## Figures and Tables

**Table 1:** Risk of bias assessment using NIH Quality Assessment of Observational Cohort and Cross-Sectional Studies.

Study	Q1	Q2	Q3	Q4	Q5	Q6	Q7	Q8	Q9	Q10	Q11	Q12	Q13	Q14
Butryn, 2019 [34]	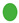	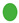	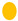	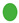	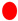	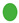	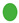	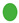	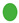	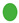	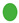	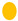	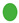	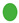
Peven, 2020 [35]	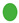	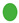	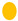	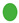	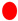	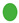	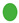	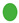	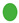	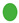	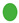	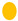	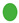	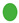
Quintero, 2018 [38]	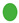	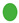	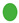	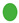	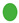	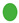	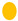	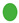	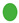	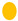	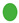	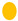	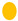	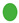
Ruegsegger, 2023 [39]	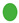	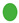	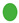	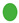	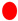	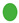	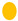	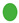	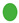	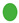	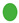	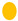	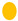	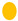
Sayuri, 2020 [36]	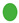	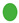	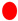	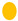	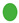	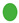	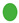	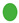	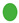	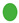	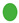	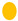	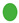	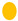
Wheeler, 2019 [40]	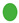	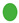	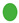	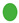	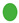	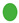	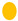	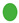	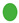	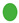	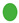	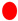	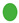	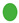
Xu, 2017 [37]	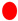	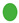	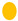	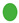	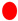	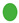	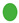	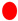	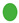	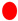	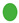	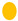	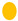	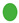

Note: low risk of bias (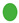); high risk of bias (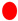); unclear risk of bias (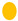).

**Table 2:** Results of physical activity interventions on executive function in adults with overweight and/or obesity from studies included.

Study	Population	Exercise	Executive Function Evaluation	Main Results
Butryn, 2019 [34]	320 male and female adults. 18–70 years old. Baseline mean BMI 35.1 kg/m^2^	6-month 16-session intervention gradually increases days and minutes of moderate-to-vigorous PA. Ultimate goal: 250 min of moderate-to-vigorous PA per week. Measured with ActiGraph GT3X accelerometer	Tower task component of Delis–Kaplan Executive Function System (D-KEFS): planning, inhibitory control, and flexibility.	Baseline moderate-to-vigorous PA negatively associated with D-KEFS time per move. D-KEFS rule violations significantly predicted moderate-to-vigorous PA at 6 months: without rule violations = 169.8 min/week, with rule violations = 127.2 min/week.
Peven, 2020 [35]	116 male and female adults 18 to 55 years old. Mean BMI 32.44 kg/m^2^	12-month intervention: Diet only. Diet + moderate exercise (150 min/week moderate-to-vigorous intensity). Diet + high exercise (250 min/week moderate-to-vigorous intensity).	Working memory (N-Back task). Cognitive flexibility (Task Switch). Inhibitory control (Stroop Color–Word Task). Reward sensitivity (Iowa Gambling Task).	Diet + high exercise: ↓ reward sensitivity. ↑ reward processing
Quintero, 2018 [38]	36 male overweight inactive adults. 18–30 years old. Mean BMI per group: Control: 28.7 kg/m^2^ Progressive resistance training (PRT): 27.8 kg/m^2^ High-Intensity Interval Training (HIIT) = 27.4 kg/m^2^ Combined (PRT + HIIT): 28.1 kg/m^2^	Four randomized trials: HIIT, PRT, HIIT + PRT, and control.	Cognitive inhibition (Stroop test). Attention capacity (d2 test).	HIIT ↑ cognitive inhibition, concentration level, items processed, and consistency domain. HIIT and PRT + HIIT ↑ cognitive inhibition and attention capacity than PRT alone. Compared to control group: HIIT group ↑ cognitive inhibition. HIIT and PRT ↑ attention capacity.
Ruegsegger, 2023 [39]	33 male and female non-glucose tolerant adults. 18–65 years. Mean BMI per group: Lean: 23.8 kg/m^2^ Non-glucose tolerant obese: 32.3 kg/m^2^	Single high-intensity 16-min aerobic exercise session.	Attention. Executive function. Inhibition control (Stroop test). Information processing speed (Stroop test and Trail Making Test). Working memory (Digit Span Test).	Exercise ↑ cognitive function and ↑ performance in: Inhibitory control. Working memory. Information processing speed.
Sayuri, 2020 [36]	20 male sedentary young adults. 18 to 36 years. Mean BMI 34.4 kg/m^2^	6-week intervention. Moderate-intensity continuous training (MICT), high-intensity intermittent training (HIIT), and control.	Coding subtests from BETA-III non-verbal intelligence test. Stoop Color and Word Test.	↑ EF from pre- to post-intervention in MICT and HIIT.
Wheeler, 2019 [40]	65 male and female older adults. 55 to 80 years old Mean BMI 31.1 kg/m^2^	Three conditions: Sitting (SIT): 8 h uninterrupted sitting. Exercise + sitting (EX + SIT): 1 h sitting, 30 min moderate intensity walking. Exercise + breaks (EX + BR): sitting 1 h, 30 min moderate intensity walking, sitting interrupted every 30 min with light intensity walking.	Cogstate computerized test battery including: Executive function (Groton Maze Learning Test). Psychomotor function and processing speed (Detection Test), Attention (Identification Test). Visual learning (One Card Learning Test). Working memory (One Back Test and Two Back Test).	↑ working memory in EX + SIT and EX + BR. ↑ executive function in EX + SIT.
Xu, 2017 [37]	18 overweight/obese male and female young adults. 18 to 25 years. Mean BMI 36.16 kg/m^2^.	4-week intervention program including aerobic and anaerobic exercise every day.	Executive function (Stroop Task).	↑ Stroop Task performance correlated to ↑ weight loss. ↓ reaction time interference predicted ↑ weight loss percentage.

Note: BMI: Body Mass Index; EF: Executive Function; ↑: improvement/better performance; ↓: reduced.
